# Pyopneumothorax from coinfection by *Trichomonas tenax* and *Geotrichum capitatum* in a child from China: a case report

**DOI:** 10.1186/s12879-021-06539-0

**Published:** 2021-08-20

**Authors:** Yuhui Wu, Yuanzhen Ye, Yanlan Yang, Weiguo Yang, Jiayin Lin, Ke Cao

**Affiliations:** 1 Department of Pediatric Intensive Care Unit, Shenzhen Children’s Hospital, 7019# Yitian Road, Shenzhen, 518038 Guangdong People’s Republic of China; 2 Department of Neurology, Shenzhen Children’s Hospital, 7019# Yitian Road, Shenzhen, 518038 Guangdong People’s Republic of China; 3 Department of Laboratory Medicine, Shenzhen Children’s Hospital, Shenzhen, China

**Keywords:** Pyopneumothorax, Pleural effusion, Protozoa, *Trichomonas*

## Abstract

**Background:**

*Trichomonas tenax* may appear in the oral cavity of humans due to poor dentition or oral hygiene. Pyopneumothorax is a serious complication of lower respiratory tract infections that very rarely can be caused by a trichomonad species in predisposed individuals. We report a rare case of pleurisy due to *T. tenax* with coinfection by a fungus.

**Case presentation:**

We describe a 16-year-old patient with cerebral palsy who presented with severe pyopneumothorax. *T. tenax* was identified by microscopic examination of the pleural effusion and next-generation sequencing. We also identified *Geotrichum capitatum* in the pleural effusion and bronchoalveolar lavage fluid cultures. Treatment with voriconazole and metronidazole successfully eliminated these pathogens and relieved the clinical symptoms. A literature review indicated this is the first reported case of pleurisy due to *T. tenax* with coinfection by a fungus.

**Conclusion:**

The rarity of pyopneumothorax caused by *T. tenax* coinfection with a fungus should not be overlooked in the clinic. These patients should be and treated in a timely manner.

## Background

Humans are common hosts of three different trichomonad species, the genitourinary *Trichomonas vaginalis*, the oral *T. tenax*, and the intestinal *Pentatrichomonas hominis*. *T. tenax* may appear in the human oral cavity due to poor dentition and hygiene. There are only rare reports of pulmonary infections by this species [1–7]. Pyopneumothorax is a serious complication of pulmonary infection. To our knowledge, there are only 7 reported cases of *T. tenax* infection and pleural empyema in the English-language medical literature to date [1–7]. Herein, we describe a patient with pyopneumothorax with coinfection by *T. tenax* and *Geotrichum capitatum*, a saccharomycete fungus formerly known as *Trichosporon capitatum* or *Brastochizomyces capitatus*. Our review of the characteristics of this patient’s condition and relevant literature may aid in future recognition of this condition.

## Case presentation

A 16-year-old boy with cerebral palsy who lived in a social welfare institute presented to the Pediatric Intensive Care Unit of our hospital. He had a 4-day history of persistent fever, respiratory distress, productive cough, and decreased appetite. Before admission, the local hospital administered ceftriaxone for 3 days due to a suspected bacterial infection, but the fever persisted. On physical examination and 2 h after taking ibuprofen, his temperature was 37.1 °C, heart rate was 137 beats/min, blood pressure was 137/78 mmHg, respiration rate was 42 breaths/min, and the saturation of pulse oximetry was 96% with an oxygen mask. The patient had poor oral hygiene but no lip cyanosis, decreased right lung respiratory movement, dullness on percussion and decreased breathing sounds over his right chest, but no rales. The results of an abdominal examination were unremarkable.

**Fig. 1:**
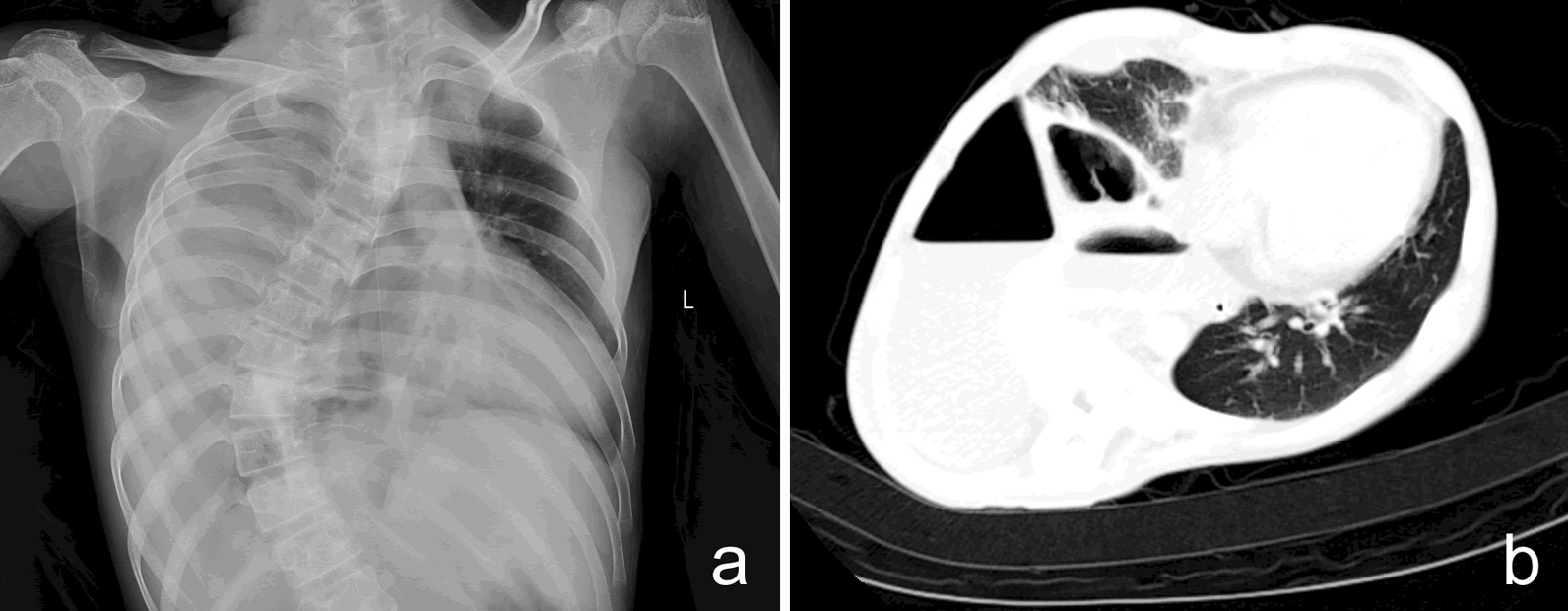
**a** Chest radiography on admission, showing massive right pleural effusion with blurred left heart border. **b** Chest CT at 2 days after admission, showing bilateral pneumonia, a large pyopneumothorax in the right pleural cavity, and an oblique fissure hydrothorax on the left

The laboratory results at admission indicated a peripheral white blood cell count of 27.15 × 10^9^/L with 80.6% neutrophils, 0.01 × 10^9^/L eosinophils, 48 g/dL hemoglobin, a hematocrit of 13.6%, and 442 × 10^9^/L platelets. The level of C-reactive protein was 323.93 mg/L and the level of procalcitonin was 40.62 ng/mL. Analysis of liver function indicated the alanine aminotransferase was 51 IU/L, aspartate aminotransferase was 95 IU/L, total bilirubin was 90.7 µmol/L, direct bilirubin was 63.2 µmol/L, albumin was 24.2 g/L, and lactate dehydrogenase was 1326 IU/L. The patient’s serum ammonia level was 181.6 μmol/L and the serum lactate level was 2.02 mmol/L. A chest radiograph and subsequent computed tomography (CT) indicated bilateral pneumonia and a large amount of pyopneumothorax in the right pleural cavity, blunting of the right costophrenic angle, oblique fissure hydrothorax on the left, and a small amount of hydrothorax in the abdominal cavity (Fig. 1).

We subsequently initiated empirical anti-infection treatment with imipenem and cilastatin and performed a thoracostomy of the right chest cavity for drainage. A macroscopic examination indicated the pleural effusion was purulent and foul-smelling. The patient’s respiratory status improved after drainage of 300 mL of the pleural effusion. We also collected bronchoalveolar lavage fluid using fiber optic bronchoscopy, and sent drainage from the empyema to the microbiology laboratory for smear and culture testing.

**Fig. 2:**
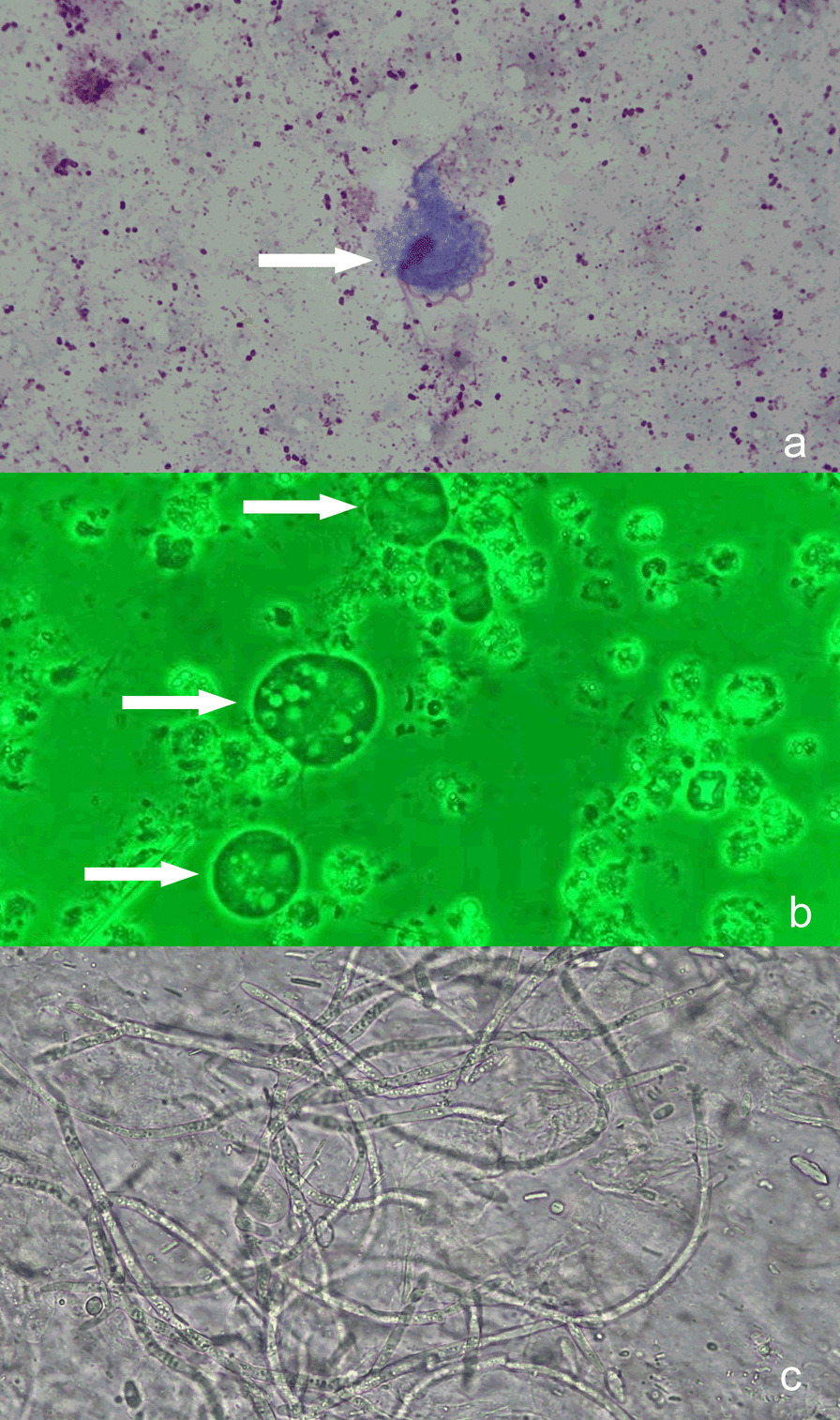
**a** Bright-field microscopy with Wright's-Giemsa staining (× 1000). **b** Phase-contrast microscopy of a sample taken during exercise (wet smear, × 1000). As indicated by the arrows, the 4 characteristic free anterior flagella, nucleus, and axial column of the *T. tenax* trophozoites were well stained. The undulating membrane is shorter than the long axis of the trophozoite, and accounts for about half of the whole trophozoite body. The slender axial column runs through the trophozoite and extends out of the body from the back, and the axostyle is relatively thick. The nucleus is located in the anterior part of the trophozoite, and has an oval shape with many chromatin granules. **c** Sputum smear, showing a large number of hyphae from *G. capitatum* (× 1000)

Microbiological analysis of the pleural fluid showed a white blood cell count of 438,000/μL with 92% polymorphonuclear leukocytes and a positive Rivalta test. Direct microscopy of a wet smear cytocentrifuged preparation created with 100 μL of the pleural fluid sediment indicated motile and flagellated organisms that were 8–10 μm in length. We made a preliminary identification of *T. tenax* based on their typical morphology, size, and wobbly and rolling motility (Fig. 2a, b) which is consistent with previous descriptions of *T. tenax* [7, 8]. The subsequent next-generation sequencing (NGS) of the pleural effusion confirmed this identification. For NGS, a 6 mL sample of pleural effusion was collected and was first enriched (~ 200 μL) using centrifugation (3000 rpm, 10 min, 4 °C). This enriched sample was used for nucleic acid extraction by Guangzhou Sagene Biotech Co. (Guangzhou, China). The metagenomic library was constructed using the protocol of the Nextera XT kit (Illumina, USA). Sequencing was performed using an Illumina Nextseq 550 DX sequencing platform. Raw data were filtered using FastQC software, human related reads were removed by aligning with a human genome reference sequence (version: GRCh38) using BWA (http://bio-bwa.sourceforge.net/) software, and then a proprietary pathogenic microbial database (including about 15,000 medical microbiological samples, optimized by Guangzhou Sagene Biotech Co., Ltd.) was used for analysis. There were 8,126,928 total raw reads, and non-human reads accounted for 44.52% of this total. Most of these reads could not be classified or mapped to any organisms in the BLAST nt database, and a small number of these reads were categorized as contaminating background reads (based on comparison to a negative control) and were filtered out. There were 178 reads of *T. tenax* and 3362 reads of *G. capitatum*. Regarding parasites and fungi, more than 10 reads in sterile body fluids (pleural effusion in this case) should be considered as “positive” according to the “Chinese expert consensus on metagenomics next-generation sequencing application on pathogen detection of infectious diseases in 2021” and other publications [9–11]. The raw data was submitted to the NCBI SRA database (accession number PRJNA738659). We also observed fungal spores and hyphae in the smear. Cultures of the pleural effusion and bronchoalveolar lavage fluid yielded *G. capitatum* (104 cfu/mL; Fig. 2c) based on mass spectrometry. There were no trichomonads in wet smears from washings of the oral cavity, no trichomonad cultures were performed, and the blood culture findings were negative.

Based on these findings, we switched the therapy to intravenous metronidazole (for *T. tenax*) and voriconazole (for *G. capitatum*). The patient’s temperature returned to normal 4 days later and *T. tenax* was not detectable after 3 days. However, one week later the boy again developed a fever. A subsequent chest tube drainage failed to resolve the effusion and a follow-up chest CT showed inadequate clearing of the pleural effusion. Thus, we performed further surgical cleaning of the pleural cavity and video-assisted thoracoscopic decortication. The patient’s temperature gradually returned to normal within 3 days after this treatment regimen. On day 18, we removed the pleural tube, and a follow-up chest x-ray showed clearing of pleural effusion. We subsequently discharged the patient on day 24, when he was in stable condition. A follow-up examination on day 30 indicated he remained in good health.

## Discussion and conclusions

Trichomonads are microscopic protozoa with four free flagella and a fifth flagellum associated with the edge of the undulating membrane. Detection is primarily by direct microscopic examination of fresh specimens, and only rarely by culture because it is usually unsuccessful. Gram staining is problematic because the fixation process destroys most of their characteristic morphologic features, making identification difficult [12]. As such, preliminary direct microscopic examinations of wet smears from freshly collected clinical samples are generally necessary for detecting these organisms, which are characterized by rapid, wobbly, and rolling motility. Results from the polymerase chain reaction and other molecular biology techniques suggest that trichomonad infections were substantially underestimated during past decades [13]. We performed direct microscopy of a wet smear cytocentrifuged preparation created with 100 μL of sediment of the pleural fluid and indentified motile and flagellated organisms that were 8–10 μm in length. We initially identified these organisms as *T. tenax* based their morphology, size, and wobbly and rolling motility, which is consistent with previous descriptions [7, 8]. We subsequently confirmed this identification using NGS.

*Trichomonas tenax* is a very important species among the trichomonads that can infect humans. It was once regarded as a harmless commensal of the oral cavity due to poor oral hygiene and dentition or periodontal disease, but this is currently debatable [8, 14]. *T. tenax* is the most common cause of trichomonad pulmonary infections, and it apparently gains entry to the lower respiratory tract by aspiration of oropharyngeal secretions. Most trichomonad infections occur in patients who are compromised by an underlying condition, such as chronic pulmonary disease, immunosuppression, human pneumocystis pneumonia, or acute respiratory distress syndrome, but infections can also occur in healthy people [15–17].

To the best of our knowledge, there are only 7 previous cases of *T. tenax* in the pleural cavity (Table 1) [1–7]. Notably, all 7 of these patients had coinfection of *T. tenax* with a bacterial species. It is thus likely that *T. tenax* is probably unable to proliferate and cause pulmonary disease or thorax empyema by itself, because it requires coinfection by an aerobic or anaerobic bacterial species as a food source. These protozoa require favorable microaerophilic conditions [18, 19]. The findings that all previous cases involving *T. tenax* infection of the pleural cavity were mixed infections with bacteria and that metronidazole relieved the clinical symptoms in these patients suggest that this trichomonad has moderate pathogenicity, and is not entirely harmless to humans.

**Table 1:** Clinical characteristics of the 8 cases of *Trichomonas tenax* associated pleural empyema

No.	Age/sex	Underlying disease(s)	Coinfection pathogen	Immunosuppressive therapy	Treatment	Outcome	References
1	87/M	Chronic pulmonary disease	Bacteria	No	MTZ, TET	Clinical improvement	[1]
2	70/M	Alcohol abuse	Bacteria	No	MTZ, CEF	Clinical improvement	[2]
3	53/M	Acromegaly rectal adenocarcinoma	Bacteria	Chemotherapy, corticotherapy, cobalt irradiation	MTZ	Clinical improvement	[7]
4	59/M	Lung adenocarcinoma	Bacteria	Corticotherapy	MTZ, GEN and CIP	Death	[3]
5	58/M	Oesophagus adenocarcinoma	Bacteria	No	MTZ, PTZ and GEN	Death	[4]
6	33/F	Heart transplantation	Bacteria	Yes	MTZ, PTZ	Clinical improvement	[5]
7	67/F	Glioblastomahigh	Bacteria	Corticotherapy	MTZ	Death	[6]
8	16/M	Cerebral palsy	Fungus	No	MTZ	Clinical improvement	Our case

The patient described here is the first reported case of pleurisy caused by infection with *T. tenax* and a fungus (*G. capitatum*) rather than a bacterium. Kurnatowska and colleagues collected a sample of 936 dental patients with different diagnoses and identified *T. tenax* in 90 cases, including 85 cases where it co-occurred with fungi. A similar finding was reported in a patient with sinusitis [20]. This result confirms that *T. tenax* may appear in humans as mixed infections with fungi, and that the prevalence of such coinfections may be underestimated [21]. The examination of our patient’s pleural effusion, bronchoalveolar lavage fluid, and blood specimens indicated no bacterial species. It is possible that *T. tenax* and *G. capitatum* were both insensitive to the initial antibiotics, and that the fungus provided a microenvironment to support the growth of *T. tenax*.

Because the rarity of pleural trichomonad infections in the clinic, they may be easily overlooked. The potential occurrence of trichomonads should therefore be considered and included in the differential diagnosis of pleural effusion in high-risk patients. Microscopic examination of a wet smear is usually successful in detection of trichomonads. The clinical application of modern molecular techniques, including the polymerase chain reaction and NGS, greatly aids in the diagnosis and identification at the species level [6, 13]. Our patient had a favorable response to metronidazole, a drug commonly used for *T. vaginalis* infections [22], and this supports our interpretation that *T. tenax* was a coinfecting agent that contributed to the patient’s pathology.

## Data Availability

The datasets used and/or analyzed during the current study are available from the corresponding author on reasonable request.

## Abbreviations

*T. tenax*: *Trichomonas tenax*
NGS: Next-generation sequencing
CT: Computed tomography
*G. capitatum*: *Geotrichum capitatum*

## References

[1] Memik F (1968). Trichomonads in pleural effusion. JAMA.

[2] Ohkura T, Suzuki N, Hashiguchi Y (1985). Invasion of the human respiratory tracts by trichomonads. Am J Trop Med Hyg.

[3] Porcheret H, Maisonneuve L, Estève V, Jagot JL, Le Pennec MP (2002). Pleural trichomoniasis due to *Trichomonas tenax*. Rev Mal Respir.

[4] Mallat H, Podglajen I, Lavarde V, Mainardi JL, Frappier J, Cornet M (2004). Molecular characterization of *Trichomonas tenax* causing pulmonary infection. J Clin Microbiol.

[5] Bellanger AP, Cabaret O, Costa JM, Foulet F, Bretagne S, Botterel F (2008). Two unusual occurrences of trichomoniasis: rapid species identification by PCR. J Clin Microbiol.

[6] Leterrier M, Morio F, Renard BT, Poirier AS, Miegeville M, Chambreuil G (2012). Trichomonads in pleural effusion: case report, literature review and utility of PCR for species identification. New Microbiol.

[7] Shiota T, Arizono N, Morimoto T, Shimatsu A, Nakao K (1998). *Trichomonas tenax* empyema in an immunocompromised patient with advanced cancer. Parasite.

[8] Marty M, Lemaitre M, Kémoun P, Morrier JJ, Monsarrat P (2017). *Trichomonas tenax* and periodontal diseases: a concise review. Parasitology.

[9] Miao Q, Ma Y, Wang Q, Pan J, Zhang Y, Jin W (2018). Microbiological diagnostic performance of metagenomic next-generation sequencing when applied to clinical practice. Clin Infect Dis.

[10] Wilson MR, Sample HA, Zorn KC, Arevalo S, Yu G, Neuhaus J (2019). Clinical metagenomic sequencing for diagnosis of meningitis and encephalitis. N Engl J Med.

[11] Brown RS, Kertiles LP, Rosenfield C, Kleinmann RE, Crigler JF (1986). Thyrotropin-receptor autoantibodies in children and young adults with Graves' disease. Am J Dis Child.

[12] Yao C, Ketzis JK (2018). Aberrant and accidental trichomonad flagellate infections: rare or underdiagnosed?. Trans R Soc Trop Med Hyg.

[13] Benabdelkader S, Andreani J, Gillet A, Terrer E, Pignoly M, Chaudet H (2019). Specific clones of *Trichomonas tenax* are associated with periodontitis. PLoS ONE.

[14] Bracamonte-Wolf C, Orrego PR, Muñoz C, Herrera D, Bravo J, Gonzalez J (2019). Observational cross-sectional study of *Trichomonas tenax* in patients with periodontal disease attending a Chilean university dental clinic. BMC Oral Health.

[15] Duboucher C, Barbier C, Beltramini A, Rona M, Ricome JL, Morel G (2007). Pulmonary superinfection by trichomonads in the course of acute respiratory distress syndrome. Lung.

[16] Thomas CF, Limper AH (2007). Current insights into the biology and pathogenesis of Pneumocystis pneumonia. Nat Rev Microbiol.

[17] Martínez-Girón R, Esteban JG, Ribas A, Doganci L (2008). Protozoa in respiratory pathology: a review. Eur Respir J.

[18] Duboucher C, Gerbod D, Noël C, Durand-Joly I, Delgado-Viscogliosi P, Leclerc C (2005). Frequency of trichomonads as coinfecting agents in Pneumocystis pneumonia. Acta Cytol.

[19] Duboucher C, Caby S, Pierce RJ, Capron M, Dei-Cas E, Viscogliosi E (2006). Trichomonads as superinfecting agents in Pneumocystis pneumonia and acute respiratory distress syndrome. J Eukaryot Microbiol.

[20] Tomás Camacho A, Pallas E, Silva J (2001). Fungal-protozoal sinusitis. Enferm Infecc Microbiol Clin.

[21] Kurnatowska AJ, Kurnatowski P (1998). Trichomonosis of the oral cavity complicated by mycosis. Parassitologia.

[22] Bala V, Chhonker YS (2018). Recent developments in anti-*Trichomonas* research: an update review. Eur J Med Chem.

